# Inflammatory Biomarkers for Assessing Treatment Response in Psoriasis: A Narrative Review

**DOI:** 10.3390/ijms27167221

**Published:** 2026-08-13

**Authors:** Julia Alicja Lewandowska, Agnieszka Owczarczyk-Saczonek, Bogusław Nedoszytko

**Affiliations:** 1 Department of Dermatology, Sexually Transmitted Diseases and Clinical Immunology, University of Warmia and Mazury, 10-719 Olsztyn, Poland; julia.lewandowska.5@doktorant.uwm.edu.pl; 2 Department of Dermatology, Venereology and Allergology, Medical University of Gdansk, 80-214 Gdansk, Poland; bned@gumed.edu.pl; 3 Department of Medical Laboratory Diagnostics-Farhenheit Biobank BBMRI.pl, Medical University of Gdansk, Debinki Street 7, 80-211 Gdansk, Poland

**Keywords:** cytokines, chemokines, acute-phase proteins, hematological indices, complete-blood-count (CBC)-derived markers, genomics, metabolomics, immunochemistry

## Abstract

Psoriasis vulgaris is a chronic immune-mediated inflammatory disease in which treatment response is assessed using clinical indices such as the Psoriasis Area and Severity Index (PASI) and Dermatology Life Quality Index (DLQI), despite their limited ability to capture systemic inflammation and underlying immunological activity. This narrative review aims to summarize current evidence on inflammatory biomarkers for monitoring treatment response and to evaluate their potential clinical utility. A structured, non-systematic literature search was performed in April–May 2026 across PubMed, Cochrane Library, Scopus, and ClinicalTrials.gov, focusing primarily on literature published during the preceding 10 years, with selected earlier studies being retained when directly relevant. Emerging data indicate that multiple biomarker domains may reflect therapeutic outcomes, including cytokines and chemokines, acute-phase proteins, complete-blood-count (CBC)-derived inflammatory indices, genetic markers, micro(mi)RNAs, metabolomic and lipidomic profiles, and tissue-based markers. These biomarkers may serve as severity-associated, baseline-predictive, pharmacodynamic, or prognostic markers, and these roles should not be interpreted interchangeably. Nevertheless, discrepancies between biomarker dynamics and clinical improvement occur, reflecting the partial dissociation between local and systemic inflammation, disease heterogeneity, and differences in response kinetics. Although inflammatory biomarkers provide a biologically grounded framework for assessing treatment response, their clinical implementation remains limited. Further large-scale, standardized studies are required to validate candidate markers and support the development of integrated, multi-omics approaches for personalized management.

## 1. Introduction

Psoriasis (PsO), a chronic inflammatory skin disease (CISD) manifests primarily as erythematous, scaly plaques in its most common form, PsO vulgaris, which accounts for over 80% of cases [1,2]. Although PsO vulgaris represents the predominant phenotype investigated in the available literature, evidence concerning inverse, palmoplantar, erythrodermic, pustular PsO and generalized pustular PsO remains limited. Therefore, unless otherwise specified, the studies discussed throughout this review primarily concern plaque PsO, although several biomarker studies included mixed populations of patients with plaque psoriasis and psoriatic arthritis, which is indicated where relevant. Beyond its cutaneous presentation driven by local microenvironment factors, including keratinocyte proliferation fueled by T helper cells (Th) 1/Th17 cells, dendritic cells, and cytokine loops such as interleukin (IL)-17, IL-21, IL-22, IFN-γ, TNF-α, IL-23, and IL-6, PsO also involves systemic inflammation marked by complete blood count (CBC)-derived indices (e.g., neutrophil-to-lymphocyte ratio (NLR), platelet-to-lymphocyte ratio (PLR), monocyte-to-lymphocyte ratio (MLR), and systemic immune-inflammation index (SII)), C-reactive protein (CRP), and other circulating cytokines, contributing to cardiometabolic risks [3,4,5,6,7].

The Psoriasis Area and Severity Index (PASI) remains the standard for therapy monitoring alongside Dermatology Life Quality Index (DLQI), yet no routine biomarkers are validated for clinical use, highlighting a critical gap in standardization and objective assessment. PASI reflects only visible skin manifestations and does not fully capture underlying immunological activity, systemic inflammation, or associated cardiovascular risk. Furthermore, it may inadequately represent disease burden in special localizations such as the scalp, nails, or genital area [8].

In contrast, inflammatory biomarkers aim to provide an objective assessment of disease activity. Discrepancies between PASI scores and biomarker levels are sometimes recognized and may arise from several factors. First, local and systemic inflammation represent partially distinct compartments. While PASI evaluates skin-limited disease, circulating biomarkers capture systemic immune activation, which may persist independently of cutaneous improvement. This divergence may be further influenced by differences in inflammatory kinetics, treatment effects, and the presence of comorbidities. Second, PsO is a heterogeneous disease, encompassing multiple phenotypes with distinct immunological profiles, meaning that PASI scores may correspond to different inflammatory states. Third, the timing of assessment is critical, as biomarkers may respond more rapidly or more slowly than clinical lesions. Finally, therapeutic interventions may differentially modulate systemic versus local inflammation, leading to further dissociation between clinical and molecular responses. 

Despite growing interest in biomarker-based assessment, no standardized inflammatory biomarkers are currently used in routine clinical practice for PsO. This reflects ongoing challenges, including lack of validation, heterogeneity of results, and limited standardization across studies. Current research points to promising genomic markers, as well as microRNA (miRNA), cytokines, chemokines, acute phase proteins, metabolomic candidates although none are ready for routine clinical use yet [9,10,11,12,13,14,15].

Cruz et al. in their work stated that quantifying PsO biomarkers from minimal blood or serum samples via high-throughput proteomics, enhances patient compliance, while establishing reliable markers involves identifying those linked to clinical outcomes like severity and response, followed by large-cohort validation of correlations, cut-offs, sensitivity, and specificity to support personalized therapy monitoring [16]. Recent advances in multi-omics research have expanded our understanding of PsO beyond conventional inflammatory pathways. It allows for the identification of disease-specific molecular signatures that may improve patient stratification, support earlier diagnosis, and provide more precise tools for monitoring therapeutic response [17]. Beside serum and histopathological assessments some evidence supports saliva as a viable diagnostic matrix in PsO with salivary IL-1β, IL-6, tumor necrosis factor (TNF)-α, and IL-17 emerging as candidate biomarkers for disease monitoring and evaluation of treatment response [18].

While several previous reviews have summarized selected biomarker classes or individual molecular pathways in PsO, the aim of the present narrative review is not to compare previous review articles but to provide a comprehensive overview of inflammatory biomarkers potentially relevant to PsO monitoring. Accordingly, this review integrates evidence across multiple biomarker domains, including circulating inflammatory mediators, acute-phase proteins, complete blood count-derived inflammatory indices, genetic biomarkers, microRNAs, metabolomic profiles, and tissue-based biomarkers. Emphasis is placed on summarizing the currently available evidence and discussing the limitations that currently prevent routine clinical implementation.

From a clinical perspective, the biomarkers discussed in this review fulfil different functions. Severity-associated biomarkers correlate with the extent or intensity of disease but do not necessarily predict treatment outcome. Baseline-predictive biomarkers are measured before treatment and are associated with the probability of response or non-response to a particular therapy. Dynamic or pharmacodynamic biomarkers change during treatment and may reflect biological response over time. Prognostic biomarkers provide information on outcomes such as treatment persistence or relapse. These categories may overlap, but they address distinct clinical questions and require separate validation.

This review aims to synthesize current evidence on biomarkers associated with disease severity, baseline prediction of therapeutic response, pharmacodynamic monitoring, and treatment-related prognosis in PsO vulgaris. Attention is given to their potential to complement established clinical indices and to the limitations that currently prevent routine clinical implementation. Accordingly, throughout this review, the assessment of treatment response is considered as a broad clinical concept encompassing baseline prediction of therapeutic outcome, dynamic monitoring during treatment, and treatment-related prognosis, rather than solely biological changes observed after treatment initiation.

## 2. Methodology

A structured literature search was performed to support this narrative review in April–May 2026. Four databases (PubMed, Cochrane Library, Scopus, and ClinicalTrials.gov) were searched using the following algorithm: (“psoriasis” OR “psoriasis vulgaris”) AND (biomarker* OR “biological marker*” OR “inflammatory marker*” OR marker*) AND (“treatment response” OR “therapy response” OR “treatment monitoring”). The aim of the literature search was to summarize current knowledge from the studies published primarily during the preceding 10 years, supplemented by selected earlier publications considered directly relevant to the review topic. The literature search was structured according to a modified PICO framework. The population (P) included adult patients with PsO vulgaris. The intervention (I) encompassed therapeutic approach with no limitations to the type of the treatment evaluated. The comparison (C) referred to changes in biomarker levels in response to treatment (baseline vs. post-treatment). The outcomes (O) focused on inflammatory biomarkers, and their role in monitoring treatment response.

Original clinical and translational studies were considered. Relevant reviews were used to provide context and to identify additional primary publications. English-language studies published up to May 2026 were included without restrictions on demographic characteristics. Selected publications were reviewed in full text, and qualitative data were extracted and synthesized by the authors. Full-text articles and abstracts were eligible when they reported a biomarker in relation to PsO severity, treatment response, treatment persistence, relapse, or a biological change during therapy. Records were excluded if they did not address PsO, did not report a biomarker-related outcome, or lacked sufficient information to identify the study population or treatment context. No minimum sample-size threshold, treatment duration, or uniform response definition was imposed because the aim was to provide a broad narrative synthesis across heterogeneous biomarker domains. This heterogeneity was considered when interpreting the findings. Because relatively few biomarker studies are available exclusively in plaque psoriasis, some cited publications included mixed populations of patients with PsO vulgaris and psoriatic arthritis. Whenever this was considered relevant for interpretation, this is explicitly indicated in the text.

**Figure 1:**
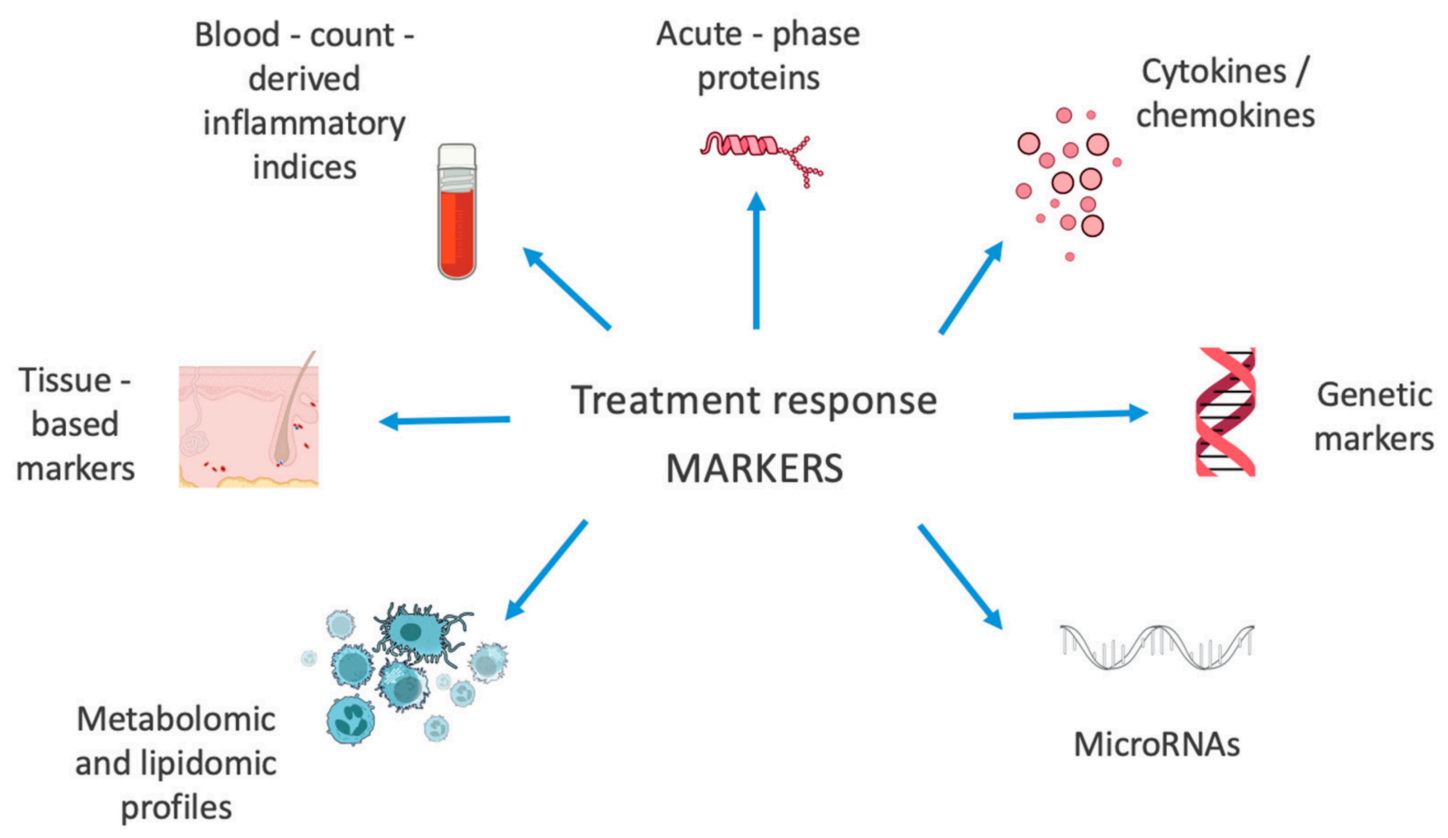
Biomarker landscape in PsO treatment response. The figure was created using Microsoft PowerPoint. Elements were adapted from NIAID Visual & Medical Arts. (2024). #77, 89, 98, 127, 253, 453, 468, 677. NIAID NIH BioArt Source.

To improve clarity and organization, the review is divided into sections (cytokines/chemokines, acute-phase/serum proteins, hematological and blood-count-derived inflammatory indices, genetic biomarkers, miRNAs, metabolomic and lipidomic biomarkers, tissue-based markers) according to major biomarker groups, within which the available evidence is critically discussed in detail. Unlike previous reviews focused predominantly on individual biomarker classes, this review integrates multiple biomarker domains within a clinically oriented framework based on their potential role in severity assessment, prediction of therapeutic response, pharmacodynamic monitoring, and prognosis. An overview of the biomarkers is presented in Figure 1. As this manuscript was designed as a narrative review, no PRISMA-based study-selection process, protocol registration, or formal risk-of-bias assessment was performed. The structured search was used to support a broad qualitative synthesis of the literature rather than an exhaustive quantitative comparison of study effects. 

## 3. Cytokines/Chemokines

Cytokines and chemokines represent a central component of the inflammatory network in PsO and have been widely investigated as biomarkers for monitoring treatment response and predicting therapeutic outcomes. Multiple studies demonstrate that specific cytokine profiles are associated with response to therapies.

A comprehensive review identified IL-6 as a marker of response to adalimumab and infliximab, IL-17, IL-21, and IL-22 for etanercept, and IL-22 also for guselkumab and ustekinumab, β-defensin-2 was linked to response to secukinumab, tofacitinib, while calprotectin to methotrexate [16]. Longitudinal analyses further suggest that dynamic cytokine profiling, especially involving IL-17A and IL-17C, may enable early prediction of treatment response as early as 3–4 weeks after therapy initiation when combined with machine learning approaches [12].

Among individual markers, IL-6 consistently emerges as one of the most clinically relevant cytokines. Its elevated baseline levels have been associated with poorer treatment outcomes and shorter drug survival, notably in patients receiving TNF-α inhibitors such as adalimumab [19,20]. In addition to IL-6, chemokines including C-C motif chemokine ligand 20 (CCL20), C-X-C motif chemokine ligand 8 (CXCL8), have demonstrated predictive value, with distinct expression patterns depending on the therapeutic target. These markers appear relevant for predicting response to anti-TNFα and anti-IL-23 therapies [21]. TNF-α itself remains a key biomarker for monitoring therapeutic efficacy. Its serum levels decrease significantly following 12 weeks biologic therapy and correlate with improvements in clinical indices such as PASI and DLQI [22]. Moreover, early reductions in TNF-α, interferon (IFN) -γ, and IL-6 during adalimumab treatment have been proposed as indicators of treatment success, whereas persistent or increasing levels may signal poor response or impending treatment failure [23]. However, not all cytokine changes directly translate into clinical outcomes, as illustrated by brodalumab therapy, where IL-17A levels decreased without consistent correlations with PASI improvement, suggesting that circulating cytokines may not fully capture therapeutic response in all settings [24].

Cytokine balance, rather than absolute levels alone, may also be critical in predicting treatment outcomes. For example, the IL-18/IL-13 ratio has been identified as a strong predictor of super-response to secukinumab, with higher IL-13 and lower IL-18 levels at baseline associated with complete skin clearance. These patients also demonstrated greater reductions in IL-18 and IL-23 during therapy, indicating that coordinated cytokine shifts may reflect deeper immunological remission [25]. In addition, thymus and activation-regulated chemokine (TARC) has been proposed as a potential biomarker of treatment response in patients treated with IL-17A inhibitors such as secukinumab, where higher levels were associated with PASI-clear status and may help guide treatment de-escalation strategies [13]. 

IL-21 represents another important cytokine with both biomarker and mechanistic relevance. Elevated levels of IL-21 and IL-21-producing T-cell subsets correlate with disease severity and decrease significantly following treatment with acitretin combined with topical therapy [4]. Evidence also highlights the importance of broader immune activation pathways. Increased nuclear factor kappa B (NF-κB) signaling, especially lipopolysaccharide (LPS)-induced NF-κBp65 phosphorylation in type-2 dendritic cells, has been identified as a predictor of non-response to adalimumab. This is accompanied by elevated IL-17+ T cells and increased numbers of cluster of differentiation (CD)83+ and IL-23-producing dendritic cells in lesional skin, suggesting that heightened baseline immune activation may underlie treatment resistance [26]. 

Cytokine dynamics during conventional therapy also provide insight into treatment mechanisms. Methotrexate has been shown to reduce pro-inflammatory cytokines such as IFN-γ, IL-2, IL-12, and IL-23 while increasing IL-4, reflecting a shift toward a less inflammatory immune profile [27]. Similarly, lower baseline plasma IL-17 levels have been associated with a better response to topical (lindioil) therapy, indicating that IL-17 may serve as a predictive biomarker of treatment outcome, although its interpretation may depend on underlying genetic background [28].

Finally, interactions between cytokine signaling and apoptosis-related pathways may further contribute to treatment response. Parallel changes in circulating sFas and sFasL levels suggest that apoptosis-related markers may reflect both therapeutic response and systemic inflammatory burden [29].

Overall, the available evidence suggests that cytokines and chemokines represent promising biomarkers of treatment response in PsO. However, the current literature is characterized by considerable heterogeneity regarding study design, patient populations, therapeutic classes, biological specimens, sampling time points, and outcome definitions. Moreover, many reported associations originate from relatively small observational or translational studies and require prospective validation before routine clinical implementation.

## 4. Acute-Phase/Serum Proteins

Acute-phase proteins and other soluble serum biomarkers constitute an important and clinically accessible group of indicators for monitoring disease activity and therapeutic response in PsO. Among these, classical inflammatory markers such as CRP have consistently demonstrated utility across multiple treatment modalities. Systemic therapies, including methotrexate and biologics such as adalimumab, lead to significant reductions in CRP levels, which correlate with improvements in PASI [14,30,31]. Notably, CRP reduction has also been observed during treatment with IL-17 and IL-23 inhibitors, including secukinumab, ixekizumab, risankizumab, and guselkumab, where it appears to be a more reliable marker than hematological indices such as NLR or PLR in longitudinal monitoring [32].

Because CRP is a nonspecific marker of systemic inflammation, obesity, metabolic syndrome, smoking, cardiovascular disease, concomitant inflammatory disorders, and psoriatic arthritis should be considered when interpreting treatment-related changes.

Beyond CRP, several acute-phase and tissue-remodeling proteins have shown promise as more specific biomarkers. YKL-40 (chitinase-3-like protein 1) is consistently elevated in PsO and correlates with disease severity, including PASI and extent of skin involvement. Its levels decrease following treatment after narrowband ultraviolet B phototherapy (NB-UVB) [33]. Similarly, serum gelsolin levels are reduced in patients with more severe disease and increase after treatment, indicating that restoration of gelsolin may reflect improvement in inflammatory status and tissue homeostasis [34].

Antimicrobial peptides and protease inhibitors also provide insight into disease activity and treatment dynamics. Human β-defensin-2 (hBD-2) has been identified as a robust biomarker, with significant reductions observed during treatment with the Janus kinase (JAK) inhibitor tofacitinib, correlating closely with PASI improvement and enabling differentiation between mild and moderate-to-severe disease [35]. Elafin (PI3), another inflammation-associated protein, has been shown to correlate positively with disease severity and to be lower in treatment responders, while persistently elevated levels may indicate treatment resistance, supporting its role in both monitoring and prognostication [36]. Squamous cell carcinoma antigen (SCCA) has demonstrated consistent correlations with PASI and body surface area (BSA) and decreases significantly (50–60%) following therapy suggesting its value as a complementary marker of disease severity and treatment response, although its interpretation may require adjustment for sex and comorbid conditions [37]. Lower baseline retinol-binding protein 4 (RBP4) and greater reductions during therapy are associated with improved PASI75 and PASI90 responses [38]. Similarly, interpretation of RBP4 requires consideration of obesity-related metabolic disturbances because circulating levels are closely linked to adiposity and insulin resistance.

Markers reflecting extracellular matrix remodeling and systemic inflammation have also been investigated. High baseline levels of microfibril-associated glycoprotein 4 (MFAP4) were associated with improved response to biologic therapy, suggesting that MFAP4 may serve as a predictive biomarker for treatment response, independent of demographic and clinical confounders. These findings originate from a broader chronic inflammatory disease cohort that included patients with PsO among other immune-mediated diseases, which may limit direct extrapolation to plaque PsO alone [39]. Calprotectin, a well-established inflammatory protein, has been linked to both disease severity and treatment outcomes, with higher baseline levels associated with greater disease activity and better response to methotrexate, as well as a potential role in predicting relapse after treatment discontinuation [40].

Tumor necrosis factor-like weak inducer of apoptosis (TWEAK) represents another relevant soluble mediator, with elevated baseline levels that decrease significantly following treatment with adalimumab or methotrexate. Importantly, changes in TWEAK correlate more strongly with PASI than with joint-related indices, indicating its relevance in both PsO vulgaris and psoriatic arthritis [41]. Because this study included patients with both PsO and psoriatic arthritis, these findings should be interpreted cautiously when extrapolated to PsO vulgaris.

Increased serum cell division cycle 42 (CDC42) levels during follow-up have been associated with better clinical outcomes (PASI75 and PASI90) in patients receiving biologic therapy, while showing an inverse relationship with disease extent and severity. These findings suggest that CDC42 may reflect resolution of inflammation and serve as a dynamic marker of treatment response [42].

Collectively, acute-phase proteins and soluble serum biomarkers provide a diverse and clinically accessible set of tools for monitoring therapeutic response in PsO. Their utility spans multiple treatment modalities, including NB-UVB, conventional systemic agents such as methotrexate, biologics such as adalimumab, secukinumab, ixekizumab, risankizumab, and guselkumab, as well as targeted small molecules like tofacitinib, highlighting their potential role in personalized treatment strategies and longitudinal disease monitoring.

Although acute-phase proteins are among the most clinically accessible biomarkers, the available evidence remains heterogeneous. Differences in treatment modalities, response definitions, follow-up duration, and analytical methods limit direct comparison between studies. Furthermore, several candidate serum biomarkers have been evaluated only in individual or exploratory cohorts and therefore require independent validation before they can be considered reliable clinical tools.

## 5. CBC-Derived Inflammatory Indices

Hematological or complete-blood-count (CBC)-derived inflammatory indices represent a readily available and cost-effective group of biomarkers that reflect systemic inflammation and have gained increasing attention in the monitoring of treatment response in PsO. Baseline hematological parameters, including neutrophil count, monocyte count, platelet count, and derived indices such as NLR, MLR, PLR, SII, and systemic inflammation response index (SIRI), are associated with disease severity. Elevated pretreatment neutrophil count, platelet count, PLR, and SII were associated with poorer persistence of conventional systemic therapies, although these findings were derived from a cohort including both patients with PsO vulgaris and psoriatic arthritis and should therefore be interpreted with caution when extrapolated to plaque psoriasis alone [43]. Methotrexate and biologics such as adalimumab lead to significant reductions in NLR and CRP, with decreases in NLR correlating positively with PASI improvement. Notably, a greater reduction in NLR has been observed in patients treated with adalimumab compared with methotrexate, suggesting that NLR may reflect both treatment efficacy and the degree of systemic inflammation control [14]. Among individual indices, NLR has emerged as both a predictive and monitoring biomarker. Lower baseline NLR values have been associated with better response to TNF-α inhibitors, including adalimumab [20]. Similarly, derived indices such as derived neutrophil-to-lymphocyte ratio (dNLR) and SIRI have shown predictive value, with higher baseline values associated with an increased likelihood of achieving a super-response during treatment with biologics such as secukinumab, ixekizumab, risankizumab, and guselkumab [6,44]. Broader panels of hematological indices, including NLR, MLR, PLR, SII, and SIRI, have been shown to decrease significantly following biologic therapy, with PLR, SII, and SIRI demonstrating the most consistent associations with clinical improvement, albeit with relatively weak correlations with PASI [45]. Long-term observations confirm that these markers, together with CRP, remain reduced over extended periods, up to 18 months, across biologic classes such as TNF-, IL-17- and IL-23 inhibitors, supporting their utility as longitudinal indicators of systemic inflammatory burden rather than precise measures of cutaneous disease activity [5]. During methotrexate therapy, SII appears to be the most responsive index, while guselkumab have been associated with consistent reductions in SIRI SII, NLR, PLR, and MLR [46,47]. Nonetheless, the utility of hematological indices remains inconsistent across studies, with some cohorts showing limited correlation between NLR or PLR and clinical response, highlighting the need for cautious interpretation. Studies of patients treated with IL-17 and IL-23 inhibitors indicate that traditional hematological ratios such as NLR and PLR may not always reliably reflect treatment response, whereas markers such as CRP and mean platelet volume (MPV) may provide more consistent information in certain cohorts [32]. Importantly, CBC-derived inflammatory indices are influenced by multiple non-psoriasis-related factors, including obesity, smoking, metabolic syndrome, cardiovascular disease, age, sex, concomitant infections, and psoriatic arthritis. Consequently, these indices should be interpreted as markers of systemic inflammatory burden rather than psoriasis-specific biomarkers.

More detailed analyses of blood-cell populations further expand the potential of hematological biomarkers. Baseline monocyte count has been identified as a predictor of early response to secukinumab, with lower monocyte counts associated with achieving low disease activity (PASI ≤ 2) at 6 months [48]. Additionally, advanced cell population data (CPD), including mean lymphocyte volume (MN-V-LY) and monocyte volume heterogeneity (SD-V-MO), have shown associations with treatment outcomes, where lower baseline MN-V-LY and early reductions in SD-V-MO correlate with better long-term response to biologic therapy [49]. Platelet–lymphocyte complexes (PLyCs) have been shown to be elevated in PsO and may predict response to adalimumab and infliximab, with higher baseline levels observed in responders and normalization occurring during therapy [50]. Similarly, circulating monocyte-derived biomarkers, including hyper-adhesive monocyte doublets, have been associated with response to apremilast, indicating that specific monocyte phenotypes may define disease endotypes more likely to benefit from phosphodiesterase 4 (PDE4) inhibition [51].

Collectively, hematological indices and blood-count-derived inflammatory markers offer a practical and integrative approach to monitoring systemic inflammation and treatment response in PsO. Their utility spans multiple therapeutic classes, including conventional systemic agents such as methotrexate, biologics targeting TNF-α (adalimumab, infliximab), IL-17 (secukinumab, ixekizumab), and IL-23 (guselkumab, risankizumab), as well as small-molecule therapies such as apremilast, highlighting their potential role in routine clinical practice and personalized treatment strategies. Nevertheless, the reported associations remain inconsistent across studies, and most evidence derives from observational cohorts using different response criteria and treatment classes. Consequently, these markers should currently be regarded as complementary indicators of systemic inflammatory burden rather than validated psoriasis-specific biomarkers.

## 6. Genetic Biomarkers

Genetic and pharmacogenetic biomarkers have emerged as important tools for predicting treatment response in PsO. Among cytokine-related polymorphisms, the *TNF-α-308 (G/A)* variant has been associated with both PsO susceptibility and biologic non-response, with the AA genotype more frequently observed in non-responders, whereas *TNFRSF1B rs1061622*, despite its association with disease risk, did not demonstrate predictive value for treatment outcomes [52]. Similarly, IL-17 pathway polymorphisms appear relevant, as IL-17F *reference SNP identifier (rs)763780* was associated with response to infliximab, and adalimumab [53]. Consistently, IL17F *rs2397084* has also been linked to improved response to methotrexate, whereas IL17A variants did not show such associations [54]. Broader analyses indicate that IL17A and IL17F polymorphisms do not universally predict response to topical therapy or NB-UVB, although they may influence treatment intensity requirements [55].

Among genetic markers, human leukocyte antigen (HLA)-C*06:02 remains the most extensively studied predictor of therapeutic response. Multiple studies indicate its association with differential response to ustekinumab, adalimumab, etanercept, infliximab [56,57,58,59]. However, findings are not fully consistent, as some data suggest that *HLA-Cw6*-positive patients may exhibit reduced response when biologics are analyzed collectively, while other studies demonstrate improved response to methotrexate and reduced adverse events in *HLA-Cw6*-positive individuals [27,60,61]. Moreover, specific allele combinations, such as *HLA-Cw06:02* positivity and *HLA-Cw01:02* negativity, together with low baseline IL-17 levels, have been associated with better response to topical therapies such as Lindioil [28].

Importantly, emerging data suggest that composite genetic signatures may outperform single biomarkers. For instance, tyrosine kinase 2 (*TYK2*) pathway-related markers, including *IL-12A*, *IL-12B*, *IL-23A*, *IL-23R*, *IL-6*, *IL-6R*, *IL-17A*, and *TNF*, have been associated with loss of treatment response, indicating that pathway-level dysregulation may better reflect therapeutic outcomes than individual polymorphisms [62].

Genome-wide association studies (GWASs) further support the role of genetic variability in treatment outcomes. Novel variants such as *rs35569429* have been linked to reduced PASI75 response to ustekinumab in combination with *HLA-C06:02* status [63]. Similarly, a GWAS in anti-TNF-treated cohorts identified multiple loci (including *rs2431355*, *rs11801616*, *rs3754679*, *rs13166823*, *rs10220768*, *rs4796752*, and *rs13045590*) associated with response to etanercept biosimilars, implicating among others genes such as *IQGAP2-F2RL2*, *SDC3*, and *IRF1-AS1* in treatment variability [64]. In the context of IL-17 pathway inhibition, variants such as *rs11649499* have been associated with complete remission following therapy with secukinumab, ixekizumab, brodalumab, and bimekizumab [65]. *ERAP1* and *ERAP2* polymorphisms, especially *ERAP2 rs2248374*, have been linked to treatment failure in secukinumab-treated patients [66]. Additionally, variants associated with response to IL-23 inhibitors, including guselkumab and risankizumab, have been identified (*rs73641950*, *rs6627462*, *rs13086445*), further supporting the relevance of pharmacogenetics in biologic therapy selection [67].

Beyond single variants, several studies highlight the importance of gene networks and immune-related pathways. Variants in genes such as for example *IL12B*, *TNFAIP3*, *CD84*, *IRAK3*, *TLR2*, *TLR5*, *TIRAP*, and *SLC12A8* have been associated with differential responses across multiple biologic classes, including TNF inhibitors (adalimumab, infliximab, etanercept), IL-17 inhibitors (secukinumab, ixekizumab, brodalumab), and IL-12/23 or IL-23 inhibitors (ustekinumab, guselkumab, risankizumab) [68,69,70]. For example, CD84 has been linked to improved response to etanercept, whereas *IL12B* and *TNFAIP3* variants have shown associations with ustekinumab response, although not consistently across all endpoints [68]. Similarly, IL-1β promoter polymorphisms (*rs752338864*) have been associated with improved response to etanercept, with elevated IL-1β levels indicating potential resistance to anti-TNF therapy [71].

In parallel, broader pharmacogenomic analyses highlight the potential contribution of genes involved in immune regulation and drug metabolism, including ABC transporters, DNMT3B, MTHFR, ANKLE1, CDKAL1, IL1B, LY96, and TLR2, and others although their clinical utility remains limited due to inconsistent replication [56,72].

Despite these advances, current evidence remains heterogeneous, and most genetic biomarkers have not yet achieved sufficient consistency for routine clinical application. Reviews consistently emphasize that, although markers such as HLA-C06:02 show the strongest evidence, variability across populations, treatment types, and study designs limits their immediate translational use, underscoring the need for large, prospective validation studies and integrated multi-omics approaches to enable true precision medicine in PsO [57,58,73]. Although numerous genetic variants have been associated with therapeutic response, most reported associations originate from individual candidate-gene studies or exploratory pharmacogenetic analyses. Consequently, these biomarkers should currently be regarded as investigational and require independent validation before routine clinical implementation.

## 7. MiRNAs

MicroRNAs have emerged as important post-transcriptional regulators of gene expression and increasingly recognized biomarkers for monitoring treatment response in PsO, reflecting both systemic and local inflammatory changes. Mechanistically, miRNAs regulate key processes such as keratinocyte proliferation, cytokine and chemokine production, and immune cell activation, thereby integrating multiple pathogenic pathways relevant to therapeutic response [74,75,76]. Evidence from studies on IL-23 inhibition indicates that circulating miRNAs may serve as sensitive indicators of therapeutic efficacy. In patients treated with the IL-23 inhibitor risankizumab, miR-200a-3p showed a positive correlation with baseline PASI, suggesting its relevance for disease severity assessment, while treatment led to reductions in IL-23, IL-1β, and IL-8 levels and an increase in regulatory T cells, supporting the link between miRNA dynamics and modulation of inflammatory pathways [77]. Complementary findings demonstrate that miR-146a and miR-155 significantly decreased following risankizumab therapy, identifying them as key circulating markers of treatment response, whereas baseline levels of miR-210 and miR-378 correlated with disease severity, indicating potential diagnostic and prognostic utility in the context of anti-IL-23 therapy [78].

In addition to IL-23-targeted therapies, miRNA profiles have also been associated with response to anti-TNF treatment. Increased expression of miR-205 in treated patients was linked to reduced TNF-α-driven inflammation, whereas miR-155 and miR-210 did not demonstrate comparable predictive value in this setting [79]. These findings highlight that individual miRNAs may exhibit treatment-specific relevance depending on the targeted inflammatory pathway.

Broader evidence from review studies confirms the central role of miRNAs in PsO pathogenesis and treatment monitoring. Multiple miRNAs, including miR-21, miR-31, miR-99a, miR-146a, miR-125b, miR-203, miR-138, and miR-155, have been consistently linked to disease severity and response to biologic therapies, supporting their potential utility as biomarkers in clinical practice [10].

Current evidence regarding miRNA biomarkers is based predominantly on relatively small exploratory studies employing different analytical platforms and normalization strategies. This methodological heterogeneity currently limits comparison across studies and prevents routine clinical implementation despite encouraging preliminary findings.

## 8. Metabolomic and Lipidomic Biomarkers

Metabolomic and lipidomic profiling has provided important insights into systemic alterations associated with PsO and their dynamic modulation during treatment, highlighting their potential role as biomarkers of therapeutic response. PsO is characterized by broad metabolic dysregulation involving amino acids, carnitines, fatty acids, lipids, and carbohydrates, which collectively reflect disease activity and systemic inflammation Treatment with the TNF inhibitor etanercept has been associated with normalization of amino acid-related metabolites, including ornithine, arginine, proline, citrulline, glycine, glutamine, threonine, and methionine, whereas the IL-17A inhibitor ixekizumab has been shown to reduce lysophosphatidylcholine (LPC) and glycerophosphocholine levels and restore lysophospholipids, dicarboxylic acids, and acylcarnitines toward physiological ranges [15].

Lipidomic analyses further support the role of metabolic biomarkers, such as pro-inflammatory lipid species, in reflecting disease activity and therapeutic response. The triglyceride-to-HDL cholesterol (TG/HDL-C) ratio has been identified as an independent predictor of long-term response to the IL-17 inhibitor secukinumab [80]. Similarly, adipokines appear to play a role in modulating treatment response in the context of IL-23 inhibition. Higher baseline leptin levels have been associated with failure to achieve PASI90 in patients treated with anti-IL-23 therapies, while visfatin levels decreased after treatment in women [81]. Because leptin concentrations primarily reflect adiposity, interpretation of this biomarker should consider obesity and metabolic syndrome as important confounding factors.

Overall, metabolomic and lipidomic data indicate that treatment response in PsO is associated with a shift toward normalization of systemic metabolic profiles, including amino acid metabolism, lipid homeostasis, and adipokine signaling. Despite their considerable biological potential, metabolomic and lipidomic biomarkers remain at an early stage of clinical development. Most available studies involve relatively limited patient cohorts and use heterogeneous analytical approaches, highlighting the need for prospective validation and methodological standardization before clinical application.

## 9. Tissue-Based Markers

Histopathological and tissue-based biomarkers provide direct insight into local inflammatory processes in PsO and their modulation during therapy, offering a complementary perspective to circulating markers. Advanced approaches such as spatial transcriptomics have demonstrated that sustained response to biologic therapy is influenced by both systemic factors, including obesity, and local epidermal inflammatory signatures associated with innate immunity and neutrophil activation. Key markers identified in lesional skin include tumor necrosis factor superfamily member 10 (TNFSF10), CXCL8, lipocalin 2 (LCN2), S100 calcium-binding protein A8 (S100A8), and S100 calcium-binding protein A9 (S100A9), alongside increased expression of IL-36α, IL-36γ, IL-1 receptor antagonist (IL-1RA), and tumor necrosis factor-related apoptosis-inducing ligand (TRAIL), suggesting that these pathways may contribute to treatment resistance and serve as potential indicators of therapeutic response [82]. Alongside with other plasma cytokines and chemokines level, skin expression CCL20 has demonstrated predictive value for anti-TNFα response, while CXCL8, IL-6, and CXCL10 may predict anti-IL-23 response [21]. Importantly, baseline characteristics of non-lesional skin also appear to carry predictive value. Expression of inflammatory mediators such as ubiquitin-specific peptidase 18 (USP18) and broader IFN/TNF-related gene signatures in clinically unaffected skin has been shown to correlate with subsequent PASI improvement in patients treated with etanercept, indicating that pre-treatment tissue immune status may influence therapeutic outcomes and could be used to anticipate PASI75 response [83]. These findings support the concept that both lesional and non-lesional compartments contribute to disease behavior and response to therapy. Expression levels of TNF-α, IL-17, and IL-23 in cutaneous biopsies have been shown to correlate positively with disease severity and treatment response, suggesting that local cytokine staining patterns may help predict the effectiveness of biologic therapies targeting these pathways [84]. Epidermal IL-36 expression may serve as a stratification marker, as higher levels are associated with better response to IL-12/23 and IL-23 inhibitors despite inconsistent correlations with other biologics, suggesting its role in identifying dominant inflammatory pathways and guiding treatment selection [85]. 

Beyond inflammatory mediators, increasing attention has been directed toward apoptosis-related pathways, which may contribute to epidermal hyperproliferation and influence treatment-induced tissue remodeling in PsO. Modulation of Fas cell surface death receptor (Fas)/Fas ligand (FasL) signaling, including changes in B-cell lymphoma-extra large (Bcl-x), Bcl-2-associated X protein (Bax), Fas, FasL, and terminal deoxynucleotidyl transferase dUTP nick end labeling (TUNEL) positivity in lesional skin, has been observed following anthralin (dithranol) therapy, indicating improved regulation of keratinocyte proliferation [86].

Proteomic analyses further expand the spectrum of tissue-related biomarkers. In patients receiving topical sequential therapy with calcipotriol and calcipotriol/betamethasone dipropionate, treatment-induced changes in proteomic pathways were observed, including reduced immune activation and modulation of lipid metabolism. Responders showed alterations in complement and coagulation cascades, whereas non-responders exhibited stronger IL-17 signaling. Candidate biomarkers associated with treatment response included Collectin subfamily member 11 (COLEC11), complement C1q A chain (C1QA), basonuclin 2 (BNC2), inter-alpha-trypsin inhibitor heavy chain 4 (ITIH4) suggesting that proteomic profiling may support monitoring of therapeutic effects and disease activity [87].

At the tissue level, changes in cutaneous miRNA expression further support their role as biomarkers of local disease activity and treatment response. Decreased expression of miR-135b following biologic therapy was associated with PASI improvement and reduced local inflammation, while additional miRNAs, including miR-133a-3p, miR-375, and miR-378a, shifted toward non-lesional expression profiles after treatment, indicating normalization of the psoriatic transcriptomic environment [88].

In patients treated with brodalumab, gene-expression signatures derived from both lesional and non-lesional skin biopsies were able to predict treatment response, with the most informative signals arising from IL-17- and peroxisome proliferator-activated receptor (PPAR)-related pathways, *HLA-D* alleles, and novel genes such as *WIF1*, *SLC44A5*, *LOC441528*, and *SAA1* [11]. These findings indicate that transcriptomic profiling captures complex disease endotypes and may enable stratification of patients prior to therapy initiation.

Tissue-based biomarkers underscore that therapeutic response in PsO is closely linked to local immune architecture, including cytokine expression, epidermal signaling pathways, and proteomic profiles. These findings suggest that integrating histopathological, transcriptomic, and proteomic data from skin biopsies may provide a more precise and mechanistic assessment of treatment response. Although tissue-based biomarkers provide important mechanistic insight into local inflammatory pathways, the available evidence remains largely derived from translational studies and relatively small patient cohorts. In addition, differences in tissue sampling, laboratory techniques, and analytical methods currently limit standardization and broader clinical applicability.

A comprehensive classification of the inflammatory biomarkers discussed in this review is presented in Table 1.

## 10. Practical Applicability and Current Limitations of Biomarker Testing

The practical value of a biomarker depends not only on its association with disease activity or treatment response but also on assay accessibility, reproducibility, cost, turnaround time, and the availability of clinically interpretable thresholds. Among the biomarkers discussed in this review, CBC parameters and CRP are the most readily accessible in routine clinical practice. CBC-derived indices can be calculated from standard hematological measurements without the need for an additional specialized assay. However, the reviewed evidence shows that their correlations with PASI and treatment response are inconsistent or relatively weak, and these indices should be interpreted as nonspecific indicators of systemic inflammatory burden rather than as independent treatment-selection tools. CRP is also widely used as a general marker of systemic inflammation and may decrease during effective systemic treatment. Nevertheless, the studies reviewed here do not support its use as a PsO-specific marker or as a substitute for clinical assessment. Its interpretation requires consideration of comorbid inflammatory conditions and the clinical context.

The remaining biomarkers generally require more specialized analytical methods. The technical ability to measure a biomarker should not be equated with validated clinical utility. Commercial availability may also differ substantially between countries and individual laboratories and was not systematically assessed in this narrative review.

For most of the biomarkers discussed, standardized cut-off values, clinically validated sensitivity and specificity estimates, and reproducible decision thresholds are unavailable. Differences in biological material, assay platforms, sampling time points, treatment classes, and definitions of response further limit direct comparison between studies. At present, specialized biomarker testing should be regarded primarily as a research tool, whereas CBC-derived indices and CRP may provide complementary, nonspecific information alongside PASI, DLQI, and clinical assessment.

## 11. Limitations of the Review

This review has several limitations. First, it was designed as a narrative review supported by a structured, non-systematic literature search and did not include a PRISMA-based selection process or a formal risk-of-bias assessment. The findings represent a qualitative synthesis rather than a quantitative comparison of biomarker performance.

Second, the underlying studies are highly heterogeneous with respect to design, patient population, treatment class, biological material, assay method, sampling time point, follow-up duration, and definition of response. Outcomes included changes in PASI, achievement of different PASI thresholds, treatment persistence, relapse, and changes in molecular markers. These differences limit direct comparisons between biomarkers.

Third, many candidate biomarkers are supported by individual, exploratory, single-center, retrospective, or post hoc studies, and external replication is frequently lacking. The review may also be affected by publication bias because positive biomarker associations are more likely to be reported than negative findings.

Fourth, the evidence discussed applies predominantly to PsO vulgaris. Its applicability to inverse, palmoplantar, erythrodermic, and pustular phenotypes remains uncertain. Some studies included mixed populations of patients with plaque PsO and psoriatic arthritis or broader cohorts of immune-mediated inflammatory diseases. Consequently, some reported biomarker associations may partly reflect systemic inflammatory processes related to joint involvement or other inflammatory conditions rather than cutaneous PsO alone.

Furthermore, because the primary objective of this narrative review was to summarize the spectrum of reported biomarkers rather than to perform a comparative appraisal of individual studies, study size and methodological quality were not systematically extracted or compared across all publications.

Finally, potential confounding factors and pre-analytical variables were not consistently reported or adjusted for across the cited studies. Consequently, associations between biomarkers and treatment outcomes cannot always be attributed to cutaneous PsO activity.

## 12. Conclusions

The available evidence indicates that inflammatory biomarkers offer a multidimensional and biologically grounded approach to assessing treatment response in PsO, extending beyond conventional clinical indices such as PASI. Across biomarker domains, including cytokines and chemokines, acute-phase proteins, hematological indices, genomic, miRNAs, metabolomic profiles, and tissue-based markers, consistent patterns emerge linking therapeutic efficacy with modulation of immune pathways, more specifically those centered on the IL-17/IL-23 axis, TNF-α signaling, and NF-κB activation. While individual biomarkers such as IL-6, TNF-α, CRP, NLR, HLA-C*06:02, or specific miRNAs demonstrate associations with treatment outcomes, the overall body of evidence supports a shift toward integrated, multi-omics approaches that capture the complexity and heterogeneity of PsO. Importantly, discrepancies between clinical improvement and biomarker dynamics highlight the need to consider both local and systemic inflammation as partially independent but complementary compartments. Despite promising results, the clinical implementation of biomarkers remains limited by heterogeneity of study designs, lack of standardized thresholds, and insufficient validation in large prospective cohorts. Therefore, future research should focus on harmonization of methodologies, longitudinal validation, and development of composite biomarker panels to enable reliable, personalized monitoring of treatment response and ultimately support precision medicine. Moreover, biomarker validation studies should account for important confounding factors, including obesity, metabolic syndrome, smoking, cardiovascular disease, age, sex, and concomitant psoriatic arthritis.

## Figures and Tables

**Table 1:** Classification and examples of inflammatory biomarkers used to monitor PsO.

Biomarker Category	Representative Biomarkers
Cytokines and chemokines	IL-2, IL-6, IL-17A, IL-17C, IL-21, IL-22, IL-23, TNF-α, IFN-γ, IL-18, IL-13, IL-4, CCL20, CXCL8, TARC, sFas, sFasL, NF-κB
Acute-phase and serum proteins	CRP, YKL-40, calprotectin, TWEAK, gelsolin, SCCA, hBD-2, PI3, MFAP4, RBP4, CDC42
CBC-derived indices	Neutrophil count, monocyte count, platelet count, NLR, PLR, MLR, SII, SIRI, dNLR, MPV, PLyC, MN-V-LY, SD-V-MO
Genetic biomarkers	HLA-C*06:02, HLA-Cw6, HLA-Cw06:02, HLA-Cw01:02, TNF-α -308 (G/A), TNFRSF1B rs1061622, IL17F rs763780, IL17F rs2397084, IL17A variants, IL17F polymorphisms, IL17A polymorphisms, IL-12A, IL-12B, IL-23A, IL-23R, IL-6, IL-6R, TNF, rs35569429, rs2431355, rs11801616, rs3754679, rs13166823, rs10220768, rs4796752, rs13045590, IQGAP2-F2RL2, SDC3, IRF1-AS1, rs11649499, ERAP1, ERAP2, rs2248374, rs73641950, rs6627462, rs13086445, TNFAIP3, CD84, IRAK3, TLR2, TLR5, TIRAP, SLC12A8, rs752338864, IL1B, ABC transporters, DNMT3B, MTHFR, ANKLE1, CDKAL1, LY96
miRNAs	miR-200a-3p, miR-146a, miR-155, miR-205, miR-210, miR-378, miR-21, miR-31, miR-99a, miR-125b, miR-203, miR-138
Metabolomic and lipidomic biomarkers	Amino acids (arginine, ornithine, citrulline, glycine, glutamine, threonine, methionine), acylcarnitines, LPC, glycerophosphocholine, dicarboxylic acids, TG/HDL-C, leptin, visfatin, proline
Tissue-based markers	TNF-α, IL-6, IL-17, IL-23, IL-36α, IL-36γ, IL-1RA, TRAIL, CXCL8, CXCL10, CCL20, LCN2, S100A8, S100A9, USP18, COLEC11, BNC2, ITIH4, IL-17 pathway genes, PPAR-related genes, HLA-D associated transcripts, WIF1, SLC44A5, SAA1, miR-135b, miR-133a-3p, miR-375, miR-378a, Bax, Bcl-x, Fas, FasL, TUNEL, LOC441528, IFN/TNF-related gene signatures

Abbreviations: ABC transporters (ATP-binding cassette transporters), ANKLE1 (ankyrin repeat and LEM domain-containing protein 1), Basonuclin 2 (BNC2), Bax (Bcl-2-associated X protein), Bcl-x (B-cell lymphoma-extra large), CBC (complete blood count), CCL20 (C-C motif chemokine ligand 20), CDC42 (cell division cycle 42), CD (cluster of differentiation), CDKAL1 (CDK5 regulatory subunit associated protein 1-like 1), C1QA (complement C1q A chain), COLEC11 (collectin subfamily member 11), CRP (C-reactive protein), CXCL8 (C-X-C motif chemokine ligand 8), dNLR (derived neutrophil-to-lymphocyte ratio), DNMT3B (DNA methyltransferase 3 beta), ERAP1 (endoplasmic reticulum aminopeptidase 1), ERAP2 (endoplasmic reticulum aminopeptidase 2), Fas (Fas cell surface death receptor), FasL (Fas ligand), hBD-2 (human β-defensin-2), HLA (human leukocyte antigen), IFN (interferon), IL (interleukin), IL1RA (IL-1 receptor antagonist), IRAK3 (IL-1 receptor-associated kinase 3), IRF1-AS1 (interferon regulatory factor 1 antisense RNA 1), ITIH4 (inter-alpha-trypsin inhibitor heavy chain 4), IQGAP2-F2RL2 (IQ motif containing GTPase activating protein 2-F2R like thrombin or trypsin receptor 2), LCN2 (lipocalin 2), LPC (lysophosphatidylcholine), LY96 (lymphocyte antigen 96), MFAP4 (microfibril-associated glycoprotein 4), miRNA (microRNA), MLR (monocyte-to-lymphocyte ratio), MMP9 (matrix metalloproteinase 9), MTHFR (methylenetetrahydrofolate reductase), MN-V-LY (mean lymphocyte volume), MPV (mean platelet volume), N-GAL (neutrophil gelatinase-associated lipocalin), NF-κB (nuclear factor kappa B), NLR (neutrophil-to-lymphocyte ratio), PI3 (peptidase inhibitor 3, elafin), PLR (platelet-to-lymphocyte ratio), PLyCs (platelet–lymphocyte complexes), PPAR (peroxisome proliferator-activated receptor), RBP4 (retinol-binding protein 4), rs (reference SNP identifier), S100A8/A9 (S100 calcium-binding protein A8/A9), SAA1 (serum amyloid A1), SCCA (squamous cell carcinoma antigen), SD-V-MO (standard deviation of monocyte volume), SII (systemic immune-inflammation index), SIRI (systemic inflammation response index), SLC44A5 (solute carrier family 44 member 5), SLC12A8 (solute carrier family 12 member 8), SDC3 (syndecan 3), TARC (thymus and activation-regulated chemokine), TG/HDL-C (triglyceride-to-high-density lipoprotein cholesterol ratio), TLR (toll-like receptor), TNF (tumor necrosis factor), TNFAIP3 (TNF alpha-induced protein 3), TNFRSF (TNF receptor superfamily member), TRAIL (TNF-related apoptosis-inducing ligand), TIRAP (TIR domain containing adaptor protein), TUNEL (terminal deoxynucleotidyl transferase dUTP nick end labeling), TWEAK (TNF-like weak inducer of apoptosis), TYK2 (tyrosine kinase 2), USP18 (ubiquitin-specific peptidase 18), WIF1 (Wnt inhibitory factor 1), YKL-40 (chitinase-3-like protein 1).

## Data Availability

No new data were created or analyzed in this study. Data sharing is not applicable to this article.
